# What distinguishes a competent doctor in medical education?

**DOI:** 10.5116/ijme.595f.b2ad

**Published:** 2017-07-20

**Authors:** Paulo Santos, Luís Alves, José Augusto Simões

**Affiliations:** 1 Department of Medicine of Community, Information and Health Decision Sciences, Faculty of Medicine, University of Porto, Portugal; 2 Life and Health Sciences Research Institute (ICVS), School of Medicine, University of Minho, Portugal; 3 Faculty of Health Sciences, University of Beira Interior, Portugal

**Keywords:** Competent doctor, medical education, Challenges of medical education, role of trainer, Portugal

## Introduction

Education is an organized process leading to the acquisition of knowledge, attitudes and skills forward to the status of proficiency. It’s a continuous and dynamic process where attaining each goal opens the perspective of new aims, enabling to structure more efficient strategies for tasks’ execution. In the medical practice, this definition flows through a pre-clinical cycle of human sciences studies, followed by a clinical course of medical training. Currently, the graduation process is complete and University recognizes the competence to practice medicine. However, the reality is inconsistent and society requires a postgraduate training program to ensure that doctors can really practice medicine. The residence time will provide the necessary skills, to become specialists.

Although Universities can play a role in post-graduation and specialization, in Portugal, as in other countries, the Ministry of Health and the official Medical Association share that responsibility. In higher education schools, professors are responsible for teaching. A peer evaluation system assesses and validates their expertise in the curricular and pedagogical fields. Out of school, the formative process of medical specialization rests on a doctor with higher experience. The supervisor guides the resident’s training program to achieve the specialist skills.

The question is about the competence of the supervisors to do it. Is there any specific profile to become a supervisor? Do they have the necessary skills for the job? Is there any kind of evaluation assessment?

In this article, we share our perspective based on Portuguese reality about the actual situation in medical education, the challenges for present and for future in medicine and their impact on education, and the redefinition of supervisors’ role to answer them.

### The supervisor

Being resident’s supervisor is an ethical duty assumed in the Hippocratic Oath and a deontological obligation of medical specialists. In this sense, we may say that all doctors are potential supervisors. However, for most of the physicians, educational activity is marginal to regular assistance practice in different areas of medicine.^1^^,^^2^ Both employers and the doctors claim for good expertise in medical field but not mandatorily in educational competencies. Medical educational programs answer to the need of good assistants but not to the need of good trainers.

The aim of the entire formative context of physicians is to assist the patient in the different phases of his life. It seeks to create good doctors, able to answer to the health needs of the population, both in anticipation or in response to disease requests. The classical paradigm of the disciple drinking from the source of his master both the medical knowledge and the posture and behavior has functioned throughout the history. It has several advantages as being extremely organized in the tasks that behooves to the teacher and those for the student, and thus creating a school in the sense that it builds a body of knowledge. However, it allows the learner to assume a passive role in learning, with little space for creativity and innovation, and thus tending to perpetuate the same kind of solutions, or even the same errors, to present and future existing problems.

If we want a continuous quality improvement in healthcare, we must break this passivity and call for new action. Only the doctor knows how to care of his patient, but to guide a younger colleague through the art of being a doctor, he’ll need more than knowing medicine: He’ll need to know how to practice medical education.

### Challenges of medical education

Since the second World War, the explosion of knowledge and global development brought, and continues to bring, new challenges where classically established strategies don’t serve as a response:^3^ the population ageing, the epidemiological transition, the sharing of knowledge with other social areas, the tasks shifting within and without medical physicians, the globalization, the wanted continuous quality improvement, and the necessary healthcare services’ humanization.

New challenges determine new practices. The 21st century asks for a doctor that goes beyond the competence to apply the objectivity of evidence-based medicine to answer people's health problems. The decision-making process integrates citizens' values, organizational systems’ needs and doctors’ ethical and deontological framework. The success of the better decision lies on the ability to share it with patients, integrating them in the responsible adoption of the prescription.

The doctor is no longer the leading actor in this frame. He accompanies the patients who choose him to be their assistant in the process,^4^ promoting health literacy, and allowing the appropriation of the necessary information for the best health decision.^5^ This deliberative model, ^6^ opposed to the classical paternalistic practice since the second half of the last century, still represents a training challenge for physicians.

Instead of the orientation for the disease’s episode, the clinical practice has changed to a patient-oriented practice. The first was slower to answer to the problems of the patients, but more systematic, rigorous and reliable. The second is faster and more satisfying for people, but more prone to errors.^7^ Likewise, communication has transferred from a preferably auditory type, based on the read or heard message, to visual strategies of decision-charts integrated in multidisciplinary care pathways. Medical art rests now on the capacity to lead the patient to appropriate himself of the necessary and sufficient information to make a conscious adoption of the medical decision. This allows to optimize adherence both to the diagnosis and to the therapeutic procedures, and to define the health objectives, individualizing the prognosis of the patient, not of the disease.

Another challenge is the modification of the clinical reasoning process. Nowadays, the diagnosis arises from the result of a probabilistic calculation through the gradual elimination of the most probable hypotheses. This problem-oriented clinical reasoning is replacing the disease-centered model where the construction of the definitive diagnosis considered a priori all diagnostic hypotheses. The model is closer to the patients in their sickness, but further from disease. In Portugal, the pre-graduation is still based predominantly in hospital settings, with limited availability to change the practice of teaching. The residents arrive to the specialization knowing how to treat the diabetes of the patient, for instance, but hardly dealing with the patient with diabetes. Efforts to change the learning processes are ongoing but not fast enough to see the results so far.

By the end of the 20th century, the results-based assessment models were incorporated into health systems, ^8^ with a significant weighting of financial variables, due to the need to rationalize the allocation of available resources. The benefit for the patient is now weighted by the costs for the individual and for the society, and by the respective willingness to pay. This is a new challenge in the medical practice, introducing the economic variables in the clinical activity, and imposing new approaches and new skills. The fundamental ethical principle of beneficence is confronted with respect for the patient's autonomy, on the one hand, and redistributive justice, on the other. In this time of explosion of knowledge, it’s difficult to keep up-to-date enough to find the balance of conciliation. Guidelines are published to fill this gap, and, despite they were built for the average of population, many doctors tend to accept them as absolute references to the individual approach.^9^ Even worse, some guidelines are presented by local authorities as normative, in the detriment of an effective patient-oriented practice,^10^ potentially more inclusive and value-generating. ^11^

These topics are challenges for the continuing medical education of all physicians. Even if we don’t completely understand them in their full extension, our commitment as supervisors obliges us to address and develop them with the residents, in a temporal perspective that overrides the residence time.

### Redefinition of the role of trainer

Doctors are then asked to be trainers in their field, enabling the resident with the experience and skills, aiming to be autonomous in the practice of their speciality. It includes training of gestures and specific tasks, development of the process of clinical reasoning, creation of a dynamic of knowledge production, practice of continuous evaluation as a key element for quality improvement processes, establishment of ethical and social communication skills, management of information and resources, leadership and humanization of care.

A qualified physician in medical education is expected to act as a model, including his / her own development. He’ll be able to identify trainees' difficulties and to plan the best strategies to deal with them, using the most appropriate methodologies. He’ll need to recognize the strengths of the residents and their weaknesses and constantly motivate them for action.

He or she must have good communication skills, be an independent evaluator, build and rebuild the formative strategies from the positive or negative aspects detected in daily practice, work in a multidisciplinary team, promote synergies and reach higher levels of efficiency.

These characteristics aren’t inborn. Supervisors should be encouraged to attend training courses, capable of filling their own perceived gaps. Examples like the courses promoted and accredited by the European Academy of Teachers in General Practice / Family Medicine (EURACT)^12^are good basis for achieving the desired improvement.^13^ They are structured in a pyramidal model of continuous formative steps^14^ based on knowledge (I know), on which skills (I know how to do), attitudes (I do) and competencies (I'm an expert) are consolidated.

In a practical approach, methods should apply to the daily situations. The evaluation forms should be systematically retrieved and analyzed and their results used as a basis for further developments.

We do an excellent exercise of medical practice when we take a clinical case of our setting and analyze it with our residents, asking for the several parts of consultation since the reception of the person up to the prognosis of the patient. Arguing about the procedures and different alternatives takes us to question our own practice. Unfortunately, the Portuguese health system reorganization of last years replaced the time of supervision for the financial payment. Supervisors actually earn some more money to take the responsibility of conducting a resident, but they have less time since their agenda is almost completely fulfilled by patients’ visits, making impossible the best proceedings. It urges a restructuration of the actual training processes, both in supervisors and residents, to reach higher levels of efficiency, making the medical education a profitable activity for health players and for organizational systems.

## Conclusion

Being competent in medical education is a necessity for physicians’ peer education and for answering to current expectations of general society. Promoting it is a responsibility of all partners in health.

Health employers have a legal duty to ensure the training of their employees through the lifetime they collaborate. The Medical Association, as seen in Portugal and in many other countries, has a statutory responsibility in defining and promoting the training needs of its professionals.

The revision of the role of supervisors is essential for the success of younger doctors’ generations and the new challenges they face nowadays. We expect that supervisors can promote innovation and development of new health solutions, at the same time that they serve as safe keepers of the values and ethical commitment that brought us to our time. There’s much work to be done, both from the point of view of systems’ organization, as from the mentality of actual supervisors, mostly accustomed to have a kind of assistant's help in their clinical activity.

Accreditation of a specific competence in medical education, with the creation of the respective College, is a strategy to encourage the change to better integration of this topic into the health agenda, recognizing it as a promoter of better health for citizens, who are the real costumers and the main beneficiaries of all this process.

### Conflict of Interest

The authors declare that they have no conflict of interest.

## References

[1] Carvalho F, Ventura T, Barroso R. Perfil de competências do orientador de formação. Revista Portuguesa de Medicina Geral e Familiar. Revista Portuguesa de Medicina Geral e Familiar. 2004;20(1):147-52.

[2] Sá AP, Teixeira-Pinto C, Veríssimo R, Vilas-Boas A, Firmino-Machado J (2015). Looking for the perfect mentor.. Acta Med Port.

[3] Sclar ED, Garau P, Carolini G (2005). The 21st century health challenge of slums and cities.. Lancet.

[4] Sackett DL. Evidence-based medicine: how to practice and teach EBM. 2nd ed. Edinburgh; New York: Churchill Livingstone; 2000.

[5] Nutbeam D (2000). Health literacy as a public health goal: a challenge for contemporary health education and communication strategies into the 21st century.. Health Promot Int.

[6] Emanuel EJ, Emanuel LL (1992). Four models of the physician-patient relationship.. JAMA.

[7] Taylor K (2009). Paternalism, participation and partnership - the evolution of patient centeredness in the consultation.. Patient Educ Couns.

[8] Henriques JS, Alexandra D (2014). [General practice and family medicine: challenges today and in the future].. Acta Med Port.

[9] Rumsfeld D (2011). Guiding the guidelines.. Lancet.

[10] Santos P, Nazaré I, Martins C, Sá L, Couto L, Hespanhol A (2015). [The Portuguese guidelines and patients values].. Acta Med Port.

[11] McCormack JP, Loewen P (2007). Adding "value" to clinical practice guidelines.. Can Fam Physician.

[12] Švab I, Allen J, Žebiene E, Petek Šter M, Windak A (2016). Training experts in family medicine teaching.. Eur J Gen Pract.

[13] Buchanan J, Maagaard R, Sammut MR, Windak A (2016). EURACT - a sustainable model for the development of teachers of General Practice/Family Medicine [GP/FM].. Educ Prim Care.

[14] Miller GE (1990). The assessment of clinical skills/competence/performance.. Acad Med.

